# 3D Printing of Air

**DOI:** 10.21203/rs.3.rs-7762382/v1

**Published:** 2025-10-28

**Authors:** Deepak Gupta, Henrique Luis Piva, Vaibhav Pal, Suihong Liu, Syed Hasan Askari Rizvi, Mecit Altan Alioglu, Yasar Ozer Yilmaz, Annie Smith, Logan Haugh, Joao Vitor Silva Robazzi, Jade Sency, Myoung Hwan Kim, Thomas Neuberger, Francesco Costanzo, Ibrahim T. Ozbolat

**Affiliations:** 1 Engineering Science and Mechanics Department, Penn State University; University Park, 16802, USA; 2 The Huck Institutes of Life Sciences, Penn State University; University Park, 16802, USA; 3 Department of Chemistry, Faculty of Philosophy, Sciences and Letters of Ribeirão Preto - University of São Paulo; Ribeirão Preto, 14040-901, Brazil; 4 Department of Chemistry, Penn State University; University Park, PA, 16802, USA; 5 Department of Nuclear Engineering, Penn State University; University Park, 16802, USA; 6 Department of Nanoscience and Nanoengineering, Istanbul Technical University; Istanbul, 34469, Turkey; 7 Biomedical Engineering Department, Penn State University; University Park, 16802, USA; 8 Department of Electrical and Computer Engineering, Federal Institute of Sao Paulo; Sertaozinho, Brazil; 9 Department of Electrical and Computer Engineering, University of Sao Paulo, Sao Carlos, Brazil; 10 Materials Research Institute, Penn State University; University Park, PA, 16802, USA; 11 Neurosurgery Department, Penn State University; University Park, PA, 16802, USA; 12 Cancer Institute, Penn State University; University Park, PA, 16802, USA

## Abstract

Historically, three-dimensional (3D) printing involved depositing tangible materials onto a substrate or within a supporting medium to create solid and porous architectures. Here, we unveil 3D printing of air, an intangible and invisible ink having ~10^9^ times less viscosity than conventional inks, to spatially pattern bubbles and fabricate freeform air channels in 3D within diverse materials. This study delves into air bubble dynamics, encompassing formation, deformation, spatiotemporal stability in non-spherical configurations, and interaction within yield stress materials. We integrate machine learning algorithms to predict air printability based on material properties, providing a framework for rational material selection. The interplay between yield stress, viscosity, and nozzle diameter facilitates the formation of stable air channels with extremely high aspect ratios (~ 4×10^4^). These insights establish a foundation for harnessing air as a printable medium and as a functional ink in applications spanning biology, optics, material science, engineering, and medicine.

Additive manufacturing refers to the layer-by-layer deposition of materials onto a substrate or within a supporting medium. Throughout its evolution, materials such as metals, ceramics, plastics, silicone, hydrogels, and microgels (μG) have been employed to fabricate three-dimensional (3D) structures. Beyond building bulk structures, considerable efforts have focused on generating open channels and prevascularized networks within these 3D structures, achieved either by casting of sacrificial templates or printing of sacrificial inks. Representative strategies include utilizing carbohydrate glass templating^1^, gallium-based engineered sacrificial capillary pumps for evacuation (ESCAPE)^2^, and rapid model guided design of organ-scale synthetic vasculature^3^, among others, wherein sacrificial inks are later evacuated to yield perfusable voids and conduits^4–8^. These techniques are often labor-intensive and cumbersome, facing multiple challenges including interfacial instability between materials^9^, solubility issues causing material shrinkage or diffusion into the matrix^7,10^, and often suffer from incomplete removal of sacrificial inks from the rough walls of high aspect ratio features. Additionally, 3D printing constantly encounters nozzle clogging issues due to drying or crosslinking of ink within the nozzle^11,12^. Recently, volumetric printing techniques offered rapid fabrication of hollow geometries without the need for support structures. However, these methods require complex instrumentation, are limited to optically transparent and compositionally homogeneous materials, and often results in partially or fully occluded high aspect ratio features due to difficulty of efficiently removing uncured resin from enclosed regions post-fabrication. Moreover, bioinks containing cells or other biologics typically exhibit high optical scattering, attenuation, and absorption, resulting in image distortions and rendering volumetric printing approaches challenging for such applications^13^.

Here, we present 3D printing of air (3DAirP) to precisely deposit air in the form of bubbles or channels at a single step, eliminating sacrificial inks entirely. Air, an invisible and intangible ink with negligible viscosity– ~10^9^ times lower than conventional sacrificial inks^5,14,15^– addresses the limitations, such as the incomplete removal of sacrificial materials, slow extrusion rates of viscous inks from a nozzle, nozzle clogging issues, and the need for labor-intensive and meticulous post-processing steps for sacrificial ink extraction, as well as significantly reduces carbon-footprint and overall fabrication cost. 3DAirP enables the deposition of air at an unprecedented speed of 2 m/s, which is 200 times faster than current embedded printing methods with speeds of ~10 mm/s^16–18^, and ~18 times faster than embedded printing in particle-hydrogel interactive system, which achieved maximum printing speed of 110 mm/s^19^. Additionally, 3DAirP demonstrates broad material compatibility including silicone-based gels, hydrogels and μG laden with biologics, such as cells or spheroids. To further support this versatility, prediction of material compatibility for 3DAirP is enabled by machine learning algorithms and supplemented with a newly designed gel tearing test that quantitatively assesses gel recovery following nozzle movement. Overall, this study advances the understanding of air–material interactions and reveals novel spatiotemporal dynamics of air bubbles within a material. For the first time, air has been employed as an invisible and intangible ink to spatially position bubbles and define channels with precise geometries within soft matter systems, thereby redefining the capabilities of 3D printing and paving the way for applications in tissue engineering, microfluidics, optics, and beyond^20^.

## Results and Discussion

**Figure 1:**
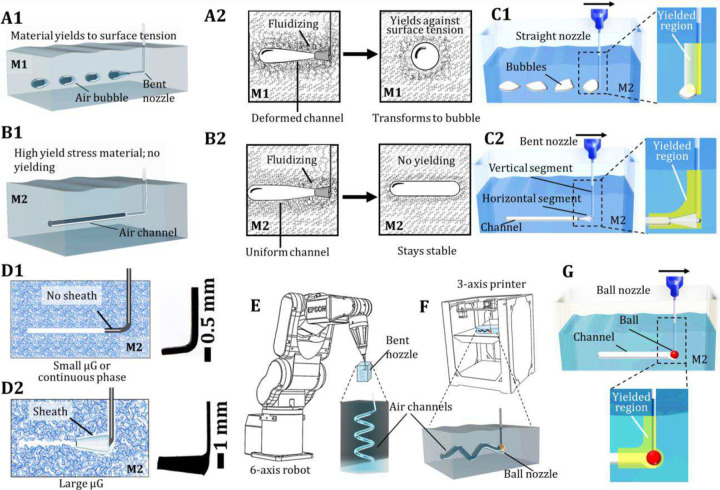
Concept of 3DAirP. (**A1**) A schematic diagram illustrates bubble formation in M1. (**A2**) Air exuding through a moving nozzle in M1 succumbs to surface tension, resulting in bubble formation. (**B1**) A schematic diagram shows channel formation in M2. (**B2**) Air exuding through a moving nozzle in M2, enabling stable channel formation due to its high yield stress, which prevents the channel from collapsing into bubbles. (**C1**) A straight nozzle translating through M2 while dispensing air results in formation of deformed bubbles. (**C2**) In contrast, a bent nozzle traversing through M2 while exuding air enables the formation of a continuous air channel by yielding the material with both horizontal and vertical segments of the nozzle. (**D1**) A bent nozzle facilitates channel formation within baths composed of either continuous phase material (such as silicon) or dispersed phase (such as microgels). (**D2**) For larger microgels, a sheath is required to locally increase the nozzle diameter, enabling stable 3DAirP without disrupting the gel matrix. (**E**) Schematics of a 6-axis robot equipped with a bent nozzle employed to achieve freeform 3DAirP. (**F**) Conventional 3D printer equipped with a ball nozzle to achieve omnidirectional 3DAirP. (**G**) A ball nozzle translating through M2 while dispensing air induces yielding of the material around the ball, resulting in formation of continuous air channels.

3DAirP leverages a fundamental understanding of the interplay between material’s yield stress, surface tension, and viscosity. In liquids, air forms spherical bubbles due to surface tension, minimizing the surface area for a given volume of air (Fig. 1A1 and fig. S1A)^21^. Although a temporary stretched bubble can be created by exuding air using a moving nozzle, the non-spherical bubble undergoes an uneven distribution of surface stress. The surrounding material, having been fluidized by the moving nozzle to a low viscosity state^22^, yields against surface tension, forcing the bubble to acquire a spherical shape (Fig. 1A2). We designate this material as material-type 1 (M1). In M1, non-spherical bubbles morph into near-spherical shape. However, unlike low viscous fluids (such as water, where bubbles rise to the surface), M1 applies enough drag force that resists the motion of bubbles^23^. The literature reports non-spherical air bubbles by combining neighboring spherical bubbles and armoring the air-liquid interface with jammed particles, which support uneven surface stresses by straddling the interface^24^. 3DAirP, however, employs a nozzle to directly inscribe air channels in 3D in a free-form manner without the need for interfacial support (Fig. 1B1, fig. S1B). This approach enables the direct formation of hollow geometries, as opposed to conventional methods that rely on material-based deposition. Channels with extremely high aspect ratios do not yield against the surface tension forces because of the material’s high yield-stress (Fig. 1B2). In our study, we achieved an exceptionally high aspect ratio of 4×10^4^ by printing a serpentine air channel measuring 20 m in length and 500 μm in diameter. This aspect ratio can be further extended as needed. We designate this high yield stress material as material-type 2 (M2). 3DAirP can be performed at an exceptional speed of 2 m/s; however, such elevated printing speeds require extremely high acceleration and deceleration settings, which may induce mechanical instabilities in the 3D printer, such as chatter and vibrational artifacts. These effects can be mitigated through improved hardware configurations and enhanced mechanical stabilization. The yield-stress materials investigated in this study include both continuous phase systems (e.g., silicone-based matrices^25^) and dispersed phase μG (e.g., gelatin-based μG). Furthermore, we identify that the performance of 3DAirP is primarily governed by three fundamental components–nozzle design, material formulation, and the interaction between nozzle and material as expounded below.

### Nozzle Design

A pivotal feature of 3DAirP is the nozzle design. When a conventional straight nozzle moves within M1 while exuding air, it creates near-spherical bubbles (figs. S2A1-A2). Whereas, within M2, a moving straight nozzle produces deformed bubbles (Fig. 1C1), with deformation aligning with the nozzle’s movement trajectory (figs. S2B1-B2). We find 3DAirP is feasible if the direction of air flow is parallel but opposite to the direction of nozzle movement. Therefore, to print an air channel, a bent nozzle is introduced. When a nozzle, bent to 90 degrees at its terminus, exudes air and is translated in the direction opposite to air flow in M2, the exuded air gets trapped within the material fluidized by the horizontal segment of the nozzle, thereby forming an air channel that follows the nozzle’s movement (Fig. 1C2).

In μG-based materials, bent nozzles with diameters ≥400 μm successfully perform 3DAirP in μG of an average size of ~50 μm (Fig. 1D1, and figs. S3A-C), whereas thicker nozzles (≥1,000 μm) are required for μG of an average size of ~85 μm (figs. S3D-E). However, the use of thicker nozzles can lead to crevice formation during movement (fig. S4), which can be mitigated at higher speeds (fig. S5). To enable 3DAirP at all speeds, a conical or cylindrical sheath is attached to the nozzle’s horizontal segment to locally increase its diameter while preventing crevice formation (Fig. 1D2, figs. S6A-B). An adjustable printhead is designed to accommodate bent nozzle and fitted on a 6-axis robot to perform 3DAirP in a free-form manner (Fig. 1E and fig. S6C). Additionally, we engineered a novel ‘ball nozzle’ configuration, featuring a spherical element affixed to the terminus of a standard blunt needle (Fig. 1F and fig. S6D). This spherical geometry introduces an extended yield region analogous to bent nozzles, thereby enabling the formation of continuous air channels (Fig. 1G). The ball nozzle supports omnidirectional printing with conventional 3D printing platforms. However, at low printing speeds (i.e., 300 mm/min), the ball nozzle tends to produce channels with rete peg patterns (fig. S7A). This patterning can be smoothened by increasing the printing speed to 2,000 mm/min, resulting in the formation of uniform cylindrical channels (fig. S7B, movie S1). This one-step channel formation via 3DAirP in μG-based systems, characterized by rough channel walls (fig. S8), overcomes limitations associated with ink-support bath interactions and tedious and incomplete ink removal from high aspect ratio geometries.

### Material Formulation

**Figure 2:**
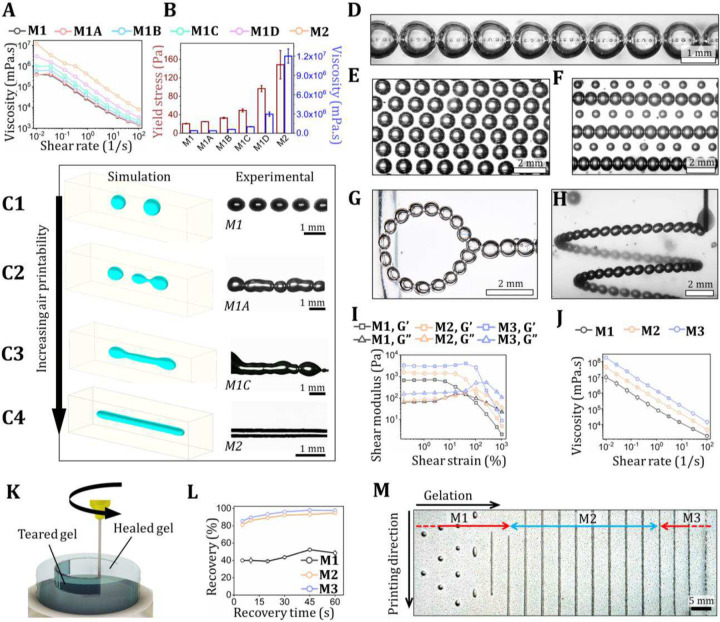
3DAirP of bubbles and channels. (**A**) Viscosity curves (under varying shear rates) and (**B**) yield stress and viscosity (at a shear rate of 0.01 s^−1^) plot of silicone-based materials from Material-type 1 (M1) to Material-type 1 (M2) along with four intermediate material formulations (M1A, M1B, M1C, and M1D) (n = 3). (**C**) Comparison of 3DAirP simulation and experiments demonstrating the gradual transformation from bubbles to channels from M1 to M2, respectively: (**C1**) Formation of separate near spherical bubbles in M1. (**C2**) Occasional merging of adjacent bubbles in M1A. (**C3**) Merged bubbles form a non-uniform channel in M1C. (**C4**) Uniform channel formation in M2. (**D**) A string of bubbles in contact with each other (without merging) printed in a silicone-based M1. (**E**) An array of uniform sized bubbles printed in a silicone-based M1. (**F**) Alternative rows of different sized bubbles achieved at different printing speeds in a silicone-based M1. Complex shaped bubble-based designs, including (**G**) a hook and (**H**) 3D spiral printed in a silicone-based M1. (**I**) Amplitude-sweep test of gelatin-based M1, M2, and M3 demonstrating their yield stress properties and increase in shear moduli from M1 to M3. (**J**) Flow curves of gelatin-based M1, M2, and M3 demonstrating their shear thinning behavior and an increase in viscosity from M1 to M3. (**K**) Schematics depicting gel tearing test, where a nozzle is translated through a material twice on the same path and the recovery of gel is measured. (**L**) Recovery (%) plotted for gelatin-based materials validating >95% recovery for M1 and M2, whereas only ~50% for M3. (**M**) Photograph depicting air printability in gelatin-based materials, demonstrating bubble printing in M1, channel printing in M2, and gel tearing in M3.

Next, the effects of material’s yield stress and viscosity on 3DAirP are investigated. In this regard, silicone-based M1 and M2 materials with four intermediate formulations (M1A, M1B, M1C, M1D) between M1 and M2 are designed and characterized for their rheological properties (Figs. 2A–B, fig. S9, Table S1). The transformation of printing bubbles into channels in these materials is explored via simulation in COMSOL Multiphysics (v6.2^26^) and experiments (Figs. 2C1–C4). Air is modeled as an incompressible Newtonian fluid (see, e.g.^27^). The non-Newtonian rheology and yield behavior of the gel is modeled as an incompressible yield-stress material with Herschel–Bulkley behavior^28^, which we regularize following the approach in^29^. We use a phase field method to track the air/gel interface^30^. Phase field methods for binary fluids use diffuse interface models to simulate mixtures of immiscible constituents, coupled with fluid constitutive laws. The model is based on a Cahn-Hilliard-system^26,31^ consisting of the following two equations:

(1) $$\left(\frac{\partial }{\partial t}+\underline{u}\cdot \nabla \right)\phi =\nabla \cdot \frac{\gamma \lambda }{\epsilon_{pf}^{2}}\nabla \psi$$

(2) $$\psi =-\nabla \cdot \epsilon_{pf}^{2}\nabla \phi +\left(\phi^{2}-1\right)\phi$$

where, *ϕ* is the dimensionless phase field variable taking values in the interval [−1, 1], the parameter *λ* (N) is the mixing energy density, $\underline{u}$ (m/s) is the velocity, *γ* is the surface tension (N/m), and *ϵ*_*pf*_ (m) is the capillary width, scaling the interface thickness. In phase field methods, a mixture of the two constituents is assumed to exist within the diffuse interface. The volume fractions of this mixture within the interface are taken to be (1 ± *ϕ*)/2. Printing an air channel in M1 results in the formation of spherical bubbles (Fig. 2C1). This result is consistent with the experimental results and indicates that surface tension, against low yield stress and viscosity, renders the non-spherical shapes infeasible. As yield stress and viscosity increases, the diminishing effects of surface tension on 3DAirP become evident, allowing the formation of non-spherical bubble shapes with increased aspect-ratio (Figs. 2C2, C3, figs. S10-S12, Movie S2). In M2, the yield stress can effectively counterbalance surface tension, stabilizing the air-printed channel (Fig. 2C4). The yield stresses, storage moduli, and viscosities of different M2 formulations are listed in Table S2. Simulation of 3DAirP in M2 with a bent nozzle at increasing printability numbers (PN: ratio of air dispense speed to nozzle speed) generates channels with progressively larger diameters, validating the 3DAirP process (figs. S13–14). The size and spacing of the bubbles in M1 can be controlled with air flow rate and nozzle speed. Spherical bubbles in proximity are printed at a slow nozzle speed (100 mm/min) (Fig. 2D), whereas elliptical bubbles distant from each other are generated at higher speeds (1,000 mm/min, fig. S15). The optimization of air flow rate and nozzle speed leads to their precise deposition forming a matrix of uniform (Fig. 2E) or alternately changing bubbles (Fig. 2F, Movie S3). Further, 2D and 3D bubble-based complex designs, such as a hook (Fig. 2G) and a growing spiral (Fig. 2H, Movie S4) are successfully achieved. It is important to note that 3DAirP of bubbles and channels is reversible in all the demonstrated materials. In other words, the printed air can be completely aspirated back through the nozzle, due to its infinitesimally slow dissolution into the material, making the material ready for subsequent printing (Movie S5). This aspect of 3DAirP conserves resources and time during optimization processes.

Upon further increasing the yield stress and viscosity beyond M2, the material’s elastic properties dominate over viscous ones. Consequently, a moving nozzle cannot fluidize the surrounding material, resulting in gel breakage and formation of a permanent crevice behind the nozzle. Here, we designate this material as Material-type 3 (M3). Transformation from M1 to M3 is characterized by changes in their rheological properties, such as storage and loss modulus, viscosity, self-healing, and self-recovery. The rheological tests results of gelatin– (Figs. 2I, J, fig. S16), GelMA– (fig. S17), and silicon– based materials (fig. S18) confirm that all materials have yield stress, are shear thinning, and exhibit self-healing and recovery properties. However, due to high yield stress of M2 and M3, vertical segments of thick nozzles create crevices behind them during their movement^16^ (fig. S4). Crevices can be alleviated in M2, but not in M3, using thinner nozzles or by increasing the translation speed of nozzles (fig. S5). The increased speed enhances the shear stress, decreases the viscosity, and improves recovery. To quantify the material recovery post nozzle translation, a new gel tearing test is designed and performed. In this test, a nozzle is translated within the materials twice along the same path at predetermined time intervals (Fig. 2K, fig. S19). The torque exerted to move the nozzle during each pass is measured, and the percentage reduction in force between iterations is calculated. More than 80% recovery is observed in gelatin-based M1 and M2 within 5 s, reaching 95% within 1 min (Fig. 2L). Whereas M3 exhibits only 40% recovery in the first 20 s, increasing to a maximum of 50% after 1 min. The transformation of the gelatin-based material from M1 to M3 is demonstrated in Fig. 2M, where bubbles are formed in M1, uniform and reproducible channels are generated in M2, and the gel is torn in M3.

**Figure 3:**
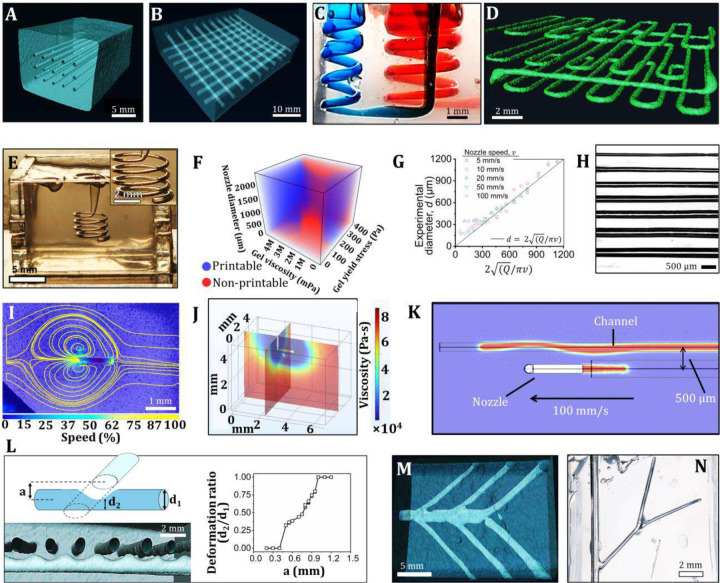
3DAirP of complex 3D structures, effects on nearby channels, and branching. **(A)** A μCT image of a 5×3 matrix of parallel air channels printed in a gelatin-based M2. **(B)** A μCT image of a matrix of air channels in two layers printed in a gelatin-based M2. **(C)** Two parallel spirals of diameter 2 and 4 mm, printed in a silicone-based M2 using a ball nozzle mounted on a conventional 3D printer, and perfused with blue and red dyes. **(D)** A μCT image of a continuous three-layered, serpentine-shaped air channel, printed in a silicone-based M2. **(E)** A semi-closed 3D continuous spiral of an air channel of diameter 4 mm and pitch 1 mm, printed in a silicone-based M2 using a 34G bent nozzle mounted on a 6-axis robot. **(F)** Prediction of air printability for different material formulations and nozzle size using machine learning models. **(G)** Theoretical and experimental comparison of channel diameter formed at different air flow rates and nozzle speeds. **(H)** Air channels of different diameters printed in a silicone-based M2. **(I)** PIV analysis demonstrates the disturbance in silicone-based M2 by moving a 34G bent nozzle inside. **(J)** Simulation results of viscosity distribution around a bent nozzle moving inside M2 at a speed of 100 mm/s. **(K)** Simulation results of printing parallel channels at a speed of 100 mm/s and gap of 500 μm, demonstrating the disturbance caused by the nozzle movement on a neighboring channel. **(L)** Controlled deformation in air channel induced by nearby perpendicular channels, creating a sinusoidal shaped channel pattern. The deformation ratio is plotted with the distance between the main channel and perpendicular channel. **(M)** A μCT image of a branched air channel in a gelatin-based M2. **(N)** Two levels of branching of air channels printed in a silicone-based M2.

With this understanding, scalable centimeter-sized structures are fabricated in gelatin-based M2 and validated through micro-computed tomography (μCT), demonstrating a 3×5 parallel matrix of uniform air channels (Fig. 3A) and a two-layered 90° matrix (Fig. 3B, fig. S20). Furthermore, complex shaped 3D perfusable channels are demonstrated, including two 3D spirals perfused with red and blue colored dyes (Fig. 3C and fig. S21A1-B2), 3D spiral printed inside a 3D crown (fig. S21C), a bubble with straight channels connected to it in a uniform circular manner resembling a spider (fig. S21D), and three-layered uniform serpentine channel made in silicone-based M2 (Fig. 3D, fig. S22, and movie S6). 3DAirP demonstrates the capability to generate semi-closed and fully enclosed channel architectures, including a hanging spiral sealed at one end (Fig. 3E, movie S7) and completely isolated channels (fig. S23A). It represents a significant advancement over conventional sacrificial printing techniques, which typically require perfusion systems to fully evacuate the sacrificial ink, and expands the design space for numerous biological applications^32,33^. Further, the channel size can be dynamically modulated during 3DAirP by adjusting nozzle speed, enabling tapered channels (fig. S23B). Moreover, the geometry of the resulting channels from 3DAirP can be further modulated post-printing by programming specific nozzle trajectories, a capability explored in this study through detailed analysis of nozzle–material interactions as expanded below.

### Nozzle-material Interactions

3DAirP and its associated dynamics are influenced by the nozzle type (bent or ball nozzle), nozzle diameter, nozzle speed, air flow rate, and rheological properties of the material. We developed machine learning algorithms, including Random Forest, XGBoost, Neural Networks, k-Nearest Neighbors, Logistic Regression, Support Vector Machines, and Naive Baye that take material’s yield stress, viscosity, and nozzle diameter as input parameters (while keeping nozzle type, nozzle speed, and air flow rate constant) and output a binary classification indicating whether a material is ‘printable’ or ‘not printable’ (Fig. 3F and fig. S24). Once a material is predicted as printable, the diameter of an air channel can be further predicted and tuned by adjusting the air flow rate and nozzle speed. For a fixed nozzle type, nozzle diameter, and material, air channels are formed at various nozzle speeds and air flow rates and compared with the theoretical diameter (Figs. 3G–H). Together, these tests can be used to predict the printability of air as well as the channel diameter.

During 3DAirP, a moving nozzle perturbs the material around it, defining the spatial limits for operations near previously printed channels. This spatial disturbance is studied using particle image velocimetry (PIV), illustrating the affected region around a bent nozzle (bottom view) (Fig. 3I and fig. S25). Simulation results validate the reduction in viscosity around the nozzle moving at a speed of 100 mm/s, attributed to shear stress (Fig. 3J). This nozzle-gel interaction deforms the channels in the vicinity of the nozzle path. Simulations of 3DAirP of a channel that is 500 μm apart from a previously printed channel causes permanent deformation of the earlier channel (Fig. 3K), corroborating experimental findings (fig. S26). Consequently, the deformation in a channel caused by printing a nearby perpendicular channel is quantified. It is observed that a channel printed perpendicular to and at a specific distance from a pre-printed channel can induce a permanent partial blockage in the latter. The degree of blockage or the deformation ratio can be precisely controlled by changing the distance between the horizontal and perpendicular channels (Fig. 3L and figs. S27A-B). Multiple perpendicular channels printed at a uniform distance from the horizontal channel render the latter with a sinusoidal pattern (Fig. 3L, fig. S27C, movie S8). Alternatively, this controlled deformation in channels can be induced by precisely extruding the bath material over a pre-printed air channel using a 3D printer with a standard blunt nozzle (figs. S27D-E). These channels with controlled degrees of blockage can be leveraged to develop disease-on-a-chip models, such as a stenosis model^34,35^.

### Branching

Branching among channels is achieved through two methods. The first method involves positioning the nozzle close to or into a pre-printed channel and creating branches from that location (movie S9). This technique is exemplified by the fabrication of perfusable branched channels in gelatin-based M2 (Fig. 3M, fig. S28A) and silicone-based M2 (figs. S28B1-B2). The second approach involves aspirating air during nozzle movement, rather than exuding it, from a pre-printed channel to create branches. Initially, an air channel, open to the atmosphere, is printed. Vacuum is then applied through a vertical blunt nozzle, which is positioned close to the pre-printed channel. The suction force removes the material between the nozzle tip and the channel and creates an air junction that marks the origination of a branch. Once the junction is formed, the nozzle is moved within the material while vacuuming air, thereby creating branches along the pre-defined path without disturbing the main channel. Branched air channels using air aspiration are demonstrated in silicone-based M2 (Fig. 3N, figs. S28C, D, and movie S10).

### Applications and Outlook

3DAirP enables the freeform fabrication and patterning of air bubbles and high aspect-ratio channels and provides new insights into their spatiotemporal stability in soft matter. These advances expand fundamental understanding of air-material interactions and open new design possibilities. It can uniquely produce both isolated bubbles and open/sealed channels within diverse soft materials in a cost-effective manner, which has significant implications across multiple scientific and engineering disciplines. In biomedical and tissue engineering domains, it can facilitate the development of perfusable vascular network^36^, microfluidic systems^37^, functional artificial organs^15^, disease models^38^, guided vascularization strategies^39^, and high-throughput drug screening platforms^40^. In materials science and soft robotics, bubble dynamics underpin the engineering of pneumatically actuated devices^41^, materials with tunable porosity for insulation^42^, and structures with targeted mechanical properties. In optics, controlled bubble behavior enables modulation of laser propagation and refractive index tuning^43^. Furthermore, extending 3DAirP to the printing of gases holds promise for environmental and industrial applications, where investigating gas entrapment and release in fluids is crucial to optimize gas-liquid interactions^44^.

**Figure 4:**
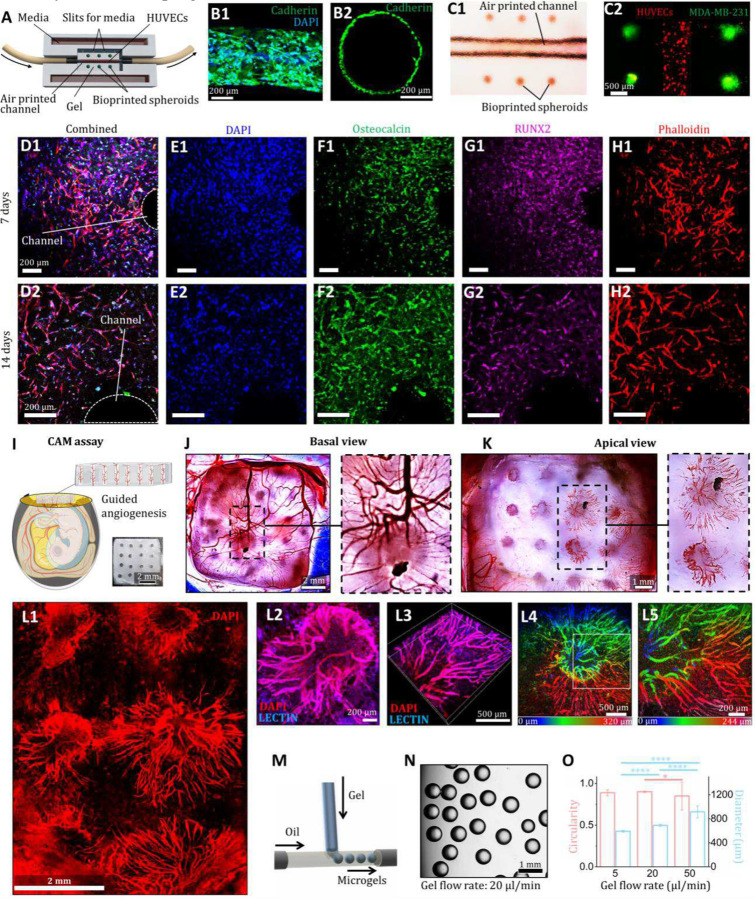
Applications of 3DAirP. **(A)** 3D model of a perfusion device with an air channel printed at the center of the gel, subsequently seeded with HUVECs. Aspiration-assisted bioprinted spheroids composed of MDA-MB-231 cells and human dermal fibroblasts are positioned on both sides of the channel. **(B1)** Formation of a HUVEC monolayer on the walls of a channel made in a GelMA-based M2. **(B2)** Cross-sectional view of the HUVEC monolayer. **(C1)** A microscopic image showing an air printed channel and aspiration-assisted bioprinted spheroids. **(C2)** Confocal image illustrating HUVECs attached to the channel walls with spheroids bioprinted on the sides after 3 days of culture under perfusion. **(D-H)** Confocal images of miR-196a-5p transfected human bone mesenchymal stem cells (hBMSCs) differentiated osteogenically within a GelMA-based M2, maintained under perfusion. Images are taken after 7 and 14 days, showing staining of osteocalcin (F1, F2), phalloidin (G1, G2), DAPI (H1, H2), RUNX2 (I1, I2), and combined staining (J1, J2). **(I)** Schematic of a chorioallantoic membrane (CAM) assay showing a construct with air printed vertical channels implanted on the CAM membrane for guided vascularization. **(J)** Basal view of construct demonstrating the penetration of the embryo’s neo-blood vessels into the channels. **(K)** Apical view of the construct with vessels following the air printed channel path and emerging out of the channels after 7 days of implantation. **(L1)** A DAPI stained stitched confocal image showing neo-vessels sprouting from multiple channels. **(L2, L3)** Magnified images with DAPI and lectin staining demonstrating vessels branching. **(L4, L5)** Depth color-coded confocal images showing vessels at different *z*-levels. **(M)** A schematic of a microfluidic device for microgel fabrication, showing the inlets for GelMA and oil and the fabrication of spherical microgels at the junction. **(N)** Microscopic image of GelMA microgels. **(O)** Plot of circularity and diameter of microgels at various gel flow rates.

Herein, we presented biomedical applications of 3DAirP using GelMA- and silicone-based materials. A custom-designed perfusion device is fabricated to facilitate the dynamic culture of tissue constructs under flow (Fig. 4A). Using this device, we fabricate (i) viable vascular-mimetic constructs, (ii) establish a model of a tumor microenvironment through the co-culture of endothelial cells, cancer cells, and fibroblasts for chemotherapeutic drug screening, and (iii) build a bone tissue analog. First of all, to validate the efficiency of the perfusion device, red dye is perfused through channels, and radial diffusion towards the spheroids composed of MDA-MB-231 and human dermal fibroblasts (HDFs), printed via aspiration-assisted bioprinting (AAB)^45^, is visualized, confirming efficient mass transport under perfusion (movie S11). Next, for drug screening, doxorubicin is perfused at varying concentrations through air printed channels embedded in GelMA-based M2 laden with MDA-MB-231 breast cancer cells, demonstrating effective diffusion and dose-dependent cytotoxicity (fig. S29). Vascular constructs are formed by seeding human umbilical vein endothelial cells (HUVECs) into air printed channels within GelMA-based M2 and maintaining them under perfusion for 7 days. Immunofluorescence staining for vascular endothelial-cadherin and DAPI confirms the formation of a HUVEC monolayer along the channel walls, mimicking blood vessels (Figs. 4B1–B2). To engineer a viable, complex tumor microenvironment, 3DAirP is integrated with AAB, featuring HUVEC-laden channels surrounded by well-aligned tumor spheroids, made with MDA-MB-231 cells and HDFs, cultured under perfusion for 3 days (Figs. 4C1–C2, movie S12). Further, 3DAirP is seamlessly integrated with extrusion-based printing to overcome geometric constraints imposed by support bath’s size and shape. For this integration, intricate geometries–such as hollow serpentine filaments–are directly printed by leveraging the favorable rheological properties of the 3DAirP materials, followed by 3DAirP to form internal channels (fig. S30). This modular integration expands the design space for fabricating complex, perfusable constructs^37^ and validates the applicability and versatility of 3DAirP system for constructing heterocellular, scalable tissue models to study tissue-specific drug delivery and disease progression^15,38^.

Furthermore, a bone tissue analog is developed using 3DAirP in a GelMA-based M2 loaded with miR-196a-5p transfected human bone mesenchymal stem cells (hBMSCs). The osteogenic differentiation of hBMSCs is analyzed following 14 days of perfusion with growth medium through the printed channels. The characteristic markers for bone tissue formation, including osteocalcin and runt-related transcription factor 2 (RUNX2), along with cell cytoskeleton and nucleus markers (phalloidin and DAPI, respectively) indicate the development of bone tissue with osteogenic cells observed after 7 and 14 days in channeled constructs (Figs. 4D1–H2), as compared to poor osteoblastic differentiation in control non-channeled constructs cultured in static conditions without perfusion (fig. S31). It validates the potential of 3DAirP in fabricating perfusable tissues to advance tissue engineering.

As the last application, we develop a GelMA-based scaffold with 16 3DAirP channels to study guided angiogenesis in an *in-ovo* model using a chicken chorioallantoic membrane (CAM) assay (Fig. 4I). The angiogenesis results after 7 days illustrate successful penetration of the embryo’s neo-vessels into the channels from the basal side and their emergence and expansion from the apical side of the channels (Figs. 4J–K), as compared to limited vessels penetration in control non-channeled scaffolds (fig. S32A-F). Confocal images with DAPI and lectin staining validate the formation of neo-vessels and their extensive sprouting through multiple channels (Fig. 4L1–L2), with a high magnification 3D image showing interconnected vasculature (Fig. 4L3) and depth color coded images demonstrating the emergence of neo-vessels from the channel at different *z*-height (Figs. 4L4–L5). Quantitative analysis using ImageJ and AngioTool (fig. S33A1–A3, B1–B3) showed that constructs with air-printed channels exhibited significantly higher total and average vessel length (fig. S33C1-C2), a significantly higher number of vessel junctions (fig. S33C3), but no significant difference in vessel-covered construct area compared to non-channeled controls (fig. S33C4). The directed growth of new blood vessels finds applications in studying therapeutic angiogenesis and controlled vasculogenesis in tissue engineering^46^. Additionally, 3DAirP of a microfluidic device is demonstrated for fabrication of μG (Fig. 4M), which are currently emerging as promising biomaterials for tissue engineering, regenerative cell therapy, and bioprinting^47–49^. GelMA microgels are prepared with this device (Fig. 4N) and their size is controlled by tuning the gel flow rate (Fig. 4O). Collectively, 3DAirP emerges as a versatile, scalable, and easy-to-implement platform for studying bubble-fluid interactions across domains and engineering perfusable, functional devices–offering a powerful alternative to conventional sacrificial printing methods.

## Supplementary Material

Supplementary Files

This is a list of supplementary files associated with this preprint. Click to download.

- SupplementaryMaterial.docx
- MovieS11.mp4
- MovieS7.mp4
- MovieS1.mp4
- MovieS8.mp4
- MovieS4.mp4
- MovieS6.mp4
- MovieS12.mp4
- MovieS2.mp4
- MovieS13.mp4
- MovieS3.mp4
- MovieS5.mp4
- MovieS9.mp4
- MovieS10.mp4

## Data Availability

All data used for machine learning, along with the source code, trained model files, and detailed results, are publicly available at the following GitHub repository: https://github.com/joaovrobazzi/AirPrintCheck. All other data are available in the main text or the supplementary materials.
